# BAG6 negatively regulates the RLR signaling pathway by targeting VISA/MAVS

**DOI:** 10.3389/fimmu.2022.972184

**Published:** 2022-08-15

**Authors:** Jing-Ping Huang, Jing Li, Yan-Ping Xiao, Liang-Guo Xu

**Affiliations:** College of Life Science, Jiangxi Normal University, Nanchang, China

**Keywords:** VISA/MAVS, BAG6, TRAF2, interferon, innate immunity

## Abstract

The virus-induced signaling adaptor protein VISA (also known as MAVS, ISP-1, Cardif) is a critical adaptor protein in the innate immune response to RNA virus infection. Upon viral infection, VISA self-aggregates to form a sizeable prion-like complex and recruits downstream signal components for signal transduction. Here, we discover that BAG6 (BCL2-associated athanogene 6, formerly BAT3 or Scythe) is an essential negative regulator in the RIG-I-like receptor signaling pathway. BAG6 inhibits the aggregation of VISA by promoting the K48-linked ubiquitination and specifically attenuates the recruitment of TRAF2 by VISA to inhibit RLR signaling. The aggregation of VISA and the interaction of VISA and TRAF2 are enhanced in BAG6-deficient cell lines after viral infection, resulting in the enhanced transcription level of downstream antiviral genes. Our research shows that BAG6 is a critical regulating factor in RIG-I/VISA-mediated innate immune response by targeting VISA.

## Introduction

The immune system is composed of innate and adaptive immunity. Innate immune is the first line of defense, which resists the invasion of microbial pathogens. Pathogen-associated molecular patterns (PAMPs) are recognized by pattern recognition receptors (PRRs) to initiate signal cascades resulting in the activation of antiviral genes such as type I interferon and proinflammatory cytokines for clearance of invading pathogens (1, 2).

The PRRs include Toll-like receptors (TLRs), RIG-I-like receptors (RLRs), DNA sensors and NOD-like receptors (3, 4). RLRs include RIG-I, MDA5, and LGP2, sensing cytoplasmic RNA viruses (3). Both RIG-I and MDA5 contain a C-terminal domain that recognizes RNA ligand, two N-terminal caspase activation and recruitment domains, which are used to transfer signals, and a central DExD/H RNA helicase domain for RNA binding and facilitating ATP hydrolysis (5, 6). RIG-I and MDA5 have a common adaptor protein VISA (also termed MAVS, IPS-1, and Cardif) (7–10) located at mitochondria. Upon RNA virus infection, the RLRs mediated antiviral signaling is activated, VISA aggregates, and forms a large prion-like polymer, which recruits downstream molecules TRAFs, TBK1, IKK complex, and cIAP1/2 for signaling (11), resulting in the activation of IRF3 and NF-KB to initiate the transcription of antiviral genes.

Previous studies have shown that RIG-I, K63-Ub4, and SNX8 induce VISA aggregation and activate VISA signaling function (11, 12). TRIM31 promotes the aggregation of VISA by K63-linked ubiquitination (13). RNF125, RNF153, AIP4, A20, SMURF2, and MARCH5 are negative regulators that inhibit VISA signaling by K48-ubiquitinating and promoting proteasome degradation of VISA except for A20 (14–18). Besides, PINK1 inhibits the aggregation and signaling of VISA (19). Whether other molecules negatively regulate VISA aggregation is unclear after viral infection.

BAG6 (BCL2-associated athanogene 6, also known as BAT3 or Scythe) is a nucleocytoplasmic shuttling protein involved in protein quality control (20, 21), identified as a ligand for NKp30 involved in antitumor immunity of NK cells (22, 23). Moreover, BAG6 forms a complex with p300 and promotes subsequent p53 acetylation, which is essential in controlling the p53 acetylation required for DNA damage responses (24, 25). In addition, BAG6 promotes the PINK1 signaling pathway and is essential for mitophagy (26). However, whether BAG6 is involved in the innate immune response is unclear. This paper discovered that BAG6 is a negative regulator in the innate immune by targeting VISA. BAG6 interacts with VISA and inhibits VISA aggregation, reducing TRAF2 recruitment. Besides, BAG6 negatively regulates RNA virus-mediated innate immune *via* promoting K48 poly-ubiquitination of VISA/TRAF2. BAG6 knockout studies suggest that BAG6 deficiency enhances the innate immune response to RNA viruses. Upon Sendai virus infection, downstream antiviral genes’ transcription level is significantly enhanced in BAG6-deficient mouse primary BMDMs cells.

## Materials and methods

### Antibodies and reagents

Anti-Flag (Sigma, F3165-0.2 MG), anti-Myc (Santa Cruz, sc-40), anti-HA (Sigma, H3663), anti-IRF3 (Santa Cruz, sc-33641; CST #4302S), anti-p-IRF3 (CST #29047, S396), anti-TBK1 (CST #3504S), anti-p-TBK1 (CST #5483S, S172), anti-P65 (CST #8242P, #12629P), anti-p-P65 (CST #3033P, S536), anti-VISA (Santa Cruz, sc-166583, sc-68881; Proteintech, 14341-1-AP), anti-TRAF2 (Santa Cruz, sc-136999), anti-Ub (Santa Cruz, sc-8017), anti-BAG6 (Santa Cruz, sc-365928; Proteintech, 26417-1-AP), anti-actin (Santa Cruz, sc-1616), anti-mouse IgG, HRP-linked (CST #7076), Alexa Fluor 555-labeled Donkey anti-Rabbit/Mouse IgG (H+L) (Beyotime Biotechnology, cat: A0453/A0460), DAPI (Solarbio, cat: C0065), Mito-Tracker Red CMXRos (Beyotime, cat: C1035). Sendai virus, VSV and VSV-GFP were donated by Dr. Hong-Bing Shu (Medical Research Institute, Wuhan University, Wuhan, China).

### Cell culture

HEK 293, HeLa, A549, NIH3T3, Vero (provided by Dr. Hong-Bing Shu, Wuhan University, China), and mouse primary BMDMs cells were cultivated in DMEM, containing 10% (vol/vol) Fetal Bovine Serum with 1% penicillin-streptomycin, with 5% CO_2_ at 37°C. All these cells were tested for no mycoplasma contamination.

### Transfection and dual-luciferase reporter assay

HEK 293 cells were cultured in a 100 mm dish, 6 or 24-wells plates, and the indicated plasmids were transferred into the cells with calcium phosphate. Sendai viruses were used to infect cells for the indicated time after 12 h, cells were harvested for analysis after virus infection, and the reporter gene experiments were performed as previously described (27, 28).

### Immunoprecipitation, native PAGE, and western blot

These experiments were performed as previously described (27, 29).

### Semi-denaturing detergent agarose gel electrophoresis (SDD-AGE)

Cells were harvested by native lysate buffer. Then, the lysates were diluted with 2 × sample buffer (1 × TBE, 20% glycerol, 4% SDS, and 0.005% bromophenol blue) and loaded with a vertical 1% agarose gel (SDD-AGE, 0.5 × TBE, 1% SDS and 1% agarose). Electrophoresis in running buffer (0.5 × TBE, 0.1% SDS) for 60 min with a constant voltage of 100 V at 4°C, the proteins were transferred to PVDF membranes with transfer buffer (0.5 × TBE) and a constant current of 0.3 A at 4°C for Western blot analysis.

### Mice and preparation of BMDMs

Wild-type mice (C57BL/6J) were purchased from Hunan Silaikejingda Experimental Animal Co., Ltd. The method of separating bone marrow cells as previously described (30). All mouse experiments were permitted by the Animal Care Committee of Jiangxi Normal University College of Life Sciences.

### Quantitative RT-PCR

The total RNA was extracted by RNA extraction Kit from Promega, then reverse transcription was performed with 1 ug of total RNA by Eastep RT Master Mix Kit from Promega. The primers for qPCR were as follows:

*β-actin* forward: GTCGTCGACAACGGCTCCGGCATG
*β-actin* reverse: ATTGTAGAAGGTGTGGTGCCAGAT
*IFN-β* forward: CTAACTGCAACCTTTCGAAGC
*IFN-β* reverse: GGAAAGAGCTGTAGTGGAGAAG
*ISG56* forward: TCATCAGGTCAAGGATAGTC
*ISG56* reverse: CCACACTGTATTTGGTGTCTAGG
*CXCL10* forward: TGACTCTAAGTGGCATTCAAGG
*CXCL10* reverse: GATTCAGACATCTCTTCTCACCC
*mGapdh* forward: TTCACCACCATGGAGAAGGC
*mGapdh* reverse: GGCATCGACTGTGGTCATGA
*mIfn-β* forward: CTGCGTTCCTGCTGTGCTTCTCCA
*mIfn-β* reverse: TTCTCCGTCATCTCCATAGGGATC
*mIsg56* forward: ACAGCAACCATGGGAGAGAATGCTG
*mIsg56* reverse: ACGTAGGCCAGGAGGTTGTGCAT
*mCxcl10* forward: CCAAGTGCTGCCGTCATTTTC
*mCxcl10* reverse: TCCCTAAGGCCCTCATTCTCA

### Plasmids

Mammalian expression plasmids, including VISA, RIG-I, RIG-I-N (1-284aa), TBK1, IKKϵ, TRAF2, TRAF5, IRF3-5D, IFN-β promoter, ISRE luciferase, ubiquitin, and its mutants K48, K63 were described previously (27, 31). pRK5-Flag/HA/Myc-hBAG6 plasmids were constructed using the following primers:

hBAG6-F-SalI (Forward): AAAAGTCGACCATGGAGCCTAATGATAGTACCAG;
hBAG6-R-NotI (Reverse): AAAAGCGGCCGCCTAAGGATCATCAGCAAAGG.

### CRISPR-Cas9 knockout.

The double-stranded oligonucleotides of human or mouse BAG6 were cloned into a lenti-CRISPR-V2 vector and transfected into HEK 293 cells with psPAX2 and pMD2G. The supernatants were harvested after 48 h and centrifuged at 3000 rpm for 15 min. The viruses were used to infect HEK 293, HeLa, NIH3T3, or BMDMs cells. The infected cells were screened with puromycin (1 μg/mL) for 7 days to obtain BAG6 deficiency monoclonal cells, except for mouse primary BMDMs. BMDMs were infected with the above viruses for 48 h and subsequently replaced with a fresh medium for viral infection.

hBAG6 gRNA: 5’-CTGTCAGGCTCCTCCACAG3’
5’-GCAAGATGATAAGAAGCTTC-3’;
mBAG6 gRNA: 5’-GCGGTACTGGCACTATCACT-3’
5’-GCCTGACAGCCTGGAGGTAC-3’.

### Plaque assays, VSV-GFP fluorescence imaging experiment, and viral infection

For plaque assays and VSV-GFP fluorescence imaging experiment, HEK 293-BAG6, HeLa-BAG6 wild, and knockout cells were infected with SeV, VSV or VSV-GFP (MOI = 0.1). After 1 h, the viruses were removed, pre-warmed PBS was used to wash the cell for twice, and cells were cultured in a fresh medium. The supernatants were harvested for the VSV-GFP fluorescence imaging experiment, and Vero cells were observed and imaged directly by fluorescence microscopy. For plaque assays, the supernatants were used to infect Vero cells for 48 h. The following steps and viral infection were performed as previously described (32).

### Fluorescent confocal microscopy

A549, HeLa cells were incubated with Mito-Tracker Red CMXRos (Beyotime, C1035) following protocols provided by the manufacturer. A549, HeLa cells were fixed with pre-warmed 4% paraformaldehyde for 15 min, permeabilized with 0.1% Triton X-100 (in PBS) for 10 min, and blocked with 1% BSA (in PBS) for 30 min. Then, cells were stained with anti-mouse VISA (Santa Cruz sc-166583, 1/250) and anti-BAG6 antibody (rabbit polyclonal, Proteintech, 26417-1-AP, 1/200) at RT for 60 min. Alexa Fluor 488-labeled Goat Anti-Mouse (green) (Beyotime Biotechnology, A0428) and Alexa Fluor 555-labeled Donkey Anti-Rabbit (red) (Beyotime Biotechnology, A0453) (diluted in 1% BSA) was added for 60 min to detect VISA and BAG6. Nuclei were stained with 10 μg/mL DAPI at RT for 15 min. Finally, images were acquired with a Leica DMi8 confocal microscopy under a ×40 objective and analyzed with Leica LAS X software.

## Result

### BAG6 interacts with VISA specifically

**Figure 1:**
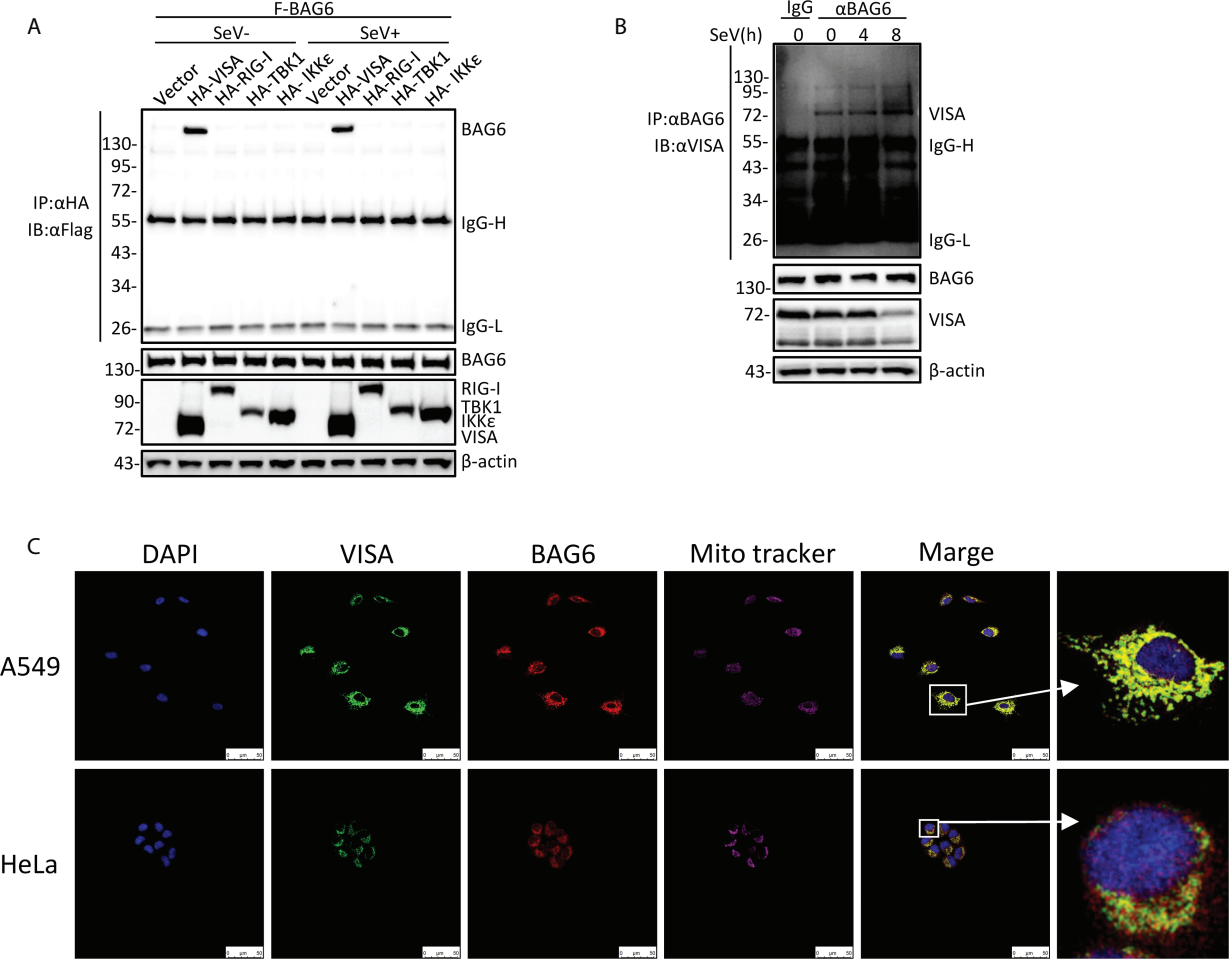
BAG6 interacts with VISA specifically. **(A)** BAG6 interacts with VISA. HEK 293 cells (2×10^6^) were transfected with indicated plasmids. 12 h after transfection, the cells were infected with SeV (MOI = 1) for 12 h or left untreated before co-immunoprecipitation and Western bolt analysis. **(B)** Endogenous BAG6 is associated with VISA. HEK 293 cells (2×10^7^) were infected with SeV (MOI = 1) for indicated times or left untreated before endogenous co-immunoprecipitation and Western bolt analysis. **(C)** A549 or HeLa cells were incubated with anti-VISA (green) and anti-BAG6 (red) antibodies. Mitochondria were stained with Mito-tracker Red CMXRos, and Nuclei were stained with DAPI. Images were acquired with a Leica DMI8 and Leica LAS X software. The size bar represents 50 μm.

To further investigate the regulatory mechanism of VISA in the RLRs-mediated antiviral signaling pathway, the yeast-two hybrid screening was performed by using VISA as the bait to screen the 293 cell cDNA expression library, trying to discover the novel partner of VISA. Multiple VISA-interacting candidate genes, including BAG6, were obtained. A further co-immunoprecipitation assay was performed in 293 cells, and the results showed that BAG6 interacts specifically with VISA (**Figure 1A**). The endogenous co-immunoprecipitation assay in HEK 293 cells consistently showed that the interaction between BAG6 and VISA was enhanced upon Sendai virus (SeV) infection (**Figure 1B**). Further immunofluorescence experiments in A549 and HeLa cells showed that BAG6 was co-localized with VISA on the outer membrane of mitochondria (**Figure 1C**), suggesting that BAG6 interacts with VISA specifically and may play a role in VISA-mediated antiviral signaling pathways.

### BAG6 negatively regulates RLR-mediated antiviral response

**Figure 2:**
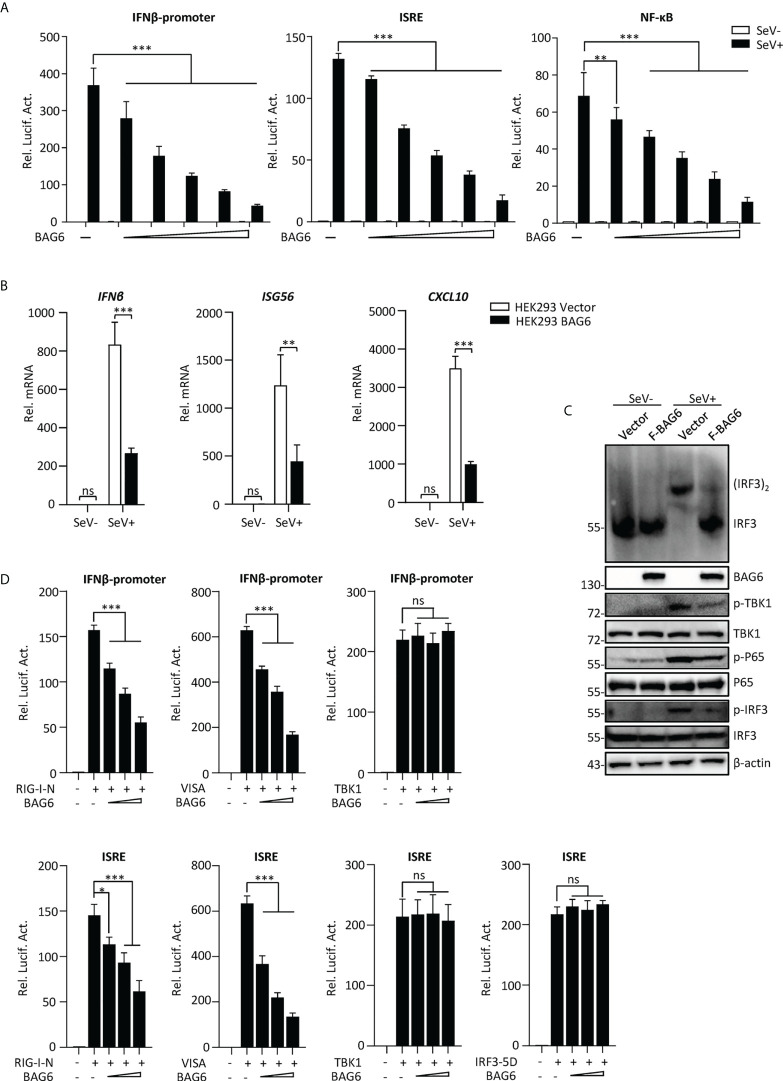
Identification of BAG6 as a negative regulator of RIG-I-mediated signaling. **(A)** BAG6 inhibits the IFN-β promoter, ISRE, and NF-κB in a dose-dependent manner. HEK 293 cells (1×10^5^) were transfected with IFN-β promoter (0.025 μg), ISRE (0.025 μg) and NF-κB (0.05 μg) reporter plasmids and BAG6 plasmids (0, 0.05, 0.1, 0.2, 0.4, 0.8 μg). Twelve hours after transfection, cells were infected with SeV (MOI = 1) for 12 h or untreated before luciferase analysis. **(B)** Overexpression of BAG6 reduces the transcription of downstream antiviral genes. HEK 293 cells were transfected with control or BAG6 plasmid for 24 h. The cells were infected with SeV (MOI = 1) for 12 h or left untreated, followed by qPCR analysis. **(C)** Overexpression of BAG6 inhibits RNA virus-induced dimerization of IRF3 and phosphorylation of TBK1, P65, and IRF3. HEK 293 cells (2×10^5^) were transfected with control and BAG6 plasmid for 24 h, and cells were left uninfected or infected with SeV (MOI = 1) for 12 h, followed by immunoblotting analysis. **(D)** BAG6 inhibits RNA virus-triggered innate immunity by targeting VISA. HEK 293 cells (3×10^5^) were transfected with IFN-β promoter (0.025 μg), ISRE (0.025 μg), BAG6 plasmids (0, 0.1, 0.2, 0.4 μg) and RIG-I-N, VISA, TBK1 or IRF3-5D (0.5 μg) for twenty h before luciferase analysis. (*p < 0.05; **p < 0.01; ***p < 0.001; ns, no significant difference).

To further investigate the role of BAG6 in RNA virus-triggered innate immunity, reporter assays were performed, and the results showed that overexpression of BAG6 in HEK 293 cells inhibited SeV-induced the activation of IFN-β promoter, ISRE, and NF-κB in a dose-dependent manner (**Figure 2A**). The results of qPCR experiments indicated that overexpression of BAG6 in HEK 293 cells reduced the transcription level of *IFNβ*, *ISG56*, and *CXCL10* induced by RNA virus (**Figure 2B**). Furthermore, overexpression of BAG6 in HEK 293 cells weakened the dimerization of IRF3 significantly and inhibited the phosphorylation of TBK1, P65, and IRF3 induced by SeV (**Figure 2C**). We then investigated BAG6’s molecular target responsible for its role in innate antiviral signaling. Reporter assay results demonstrated that BAG6 inhibited RIG-I-N and VISA-mediated activation of the IFN-β promoter and ISRE, but not the downstream TBK1 and IRF3-5D (**Figure 2D**), suggesting that BAG6 negatively regulated RNA virus-triggered innate immune cells by targeting VISA.

### The deficiency of BAG6 enhances SeV-mediated antiviral immune responses in human cell lines

**Figure 3:**
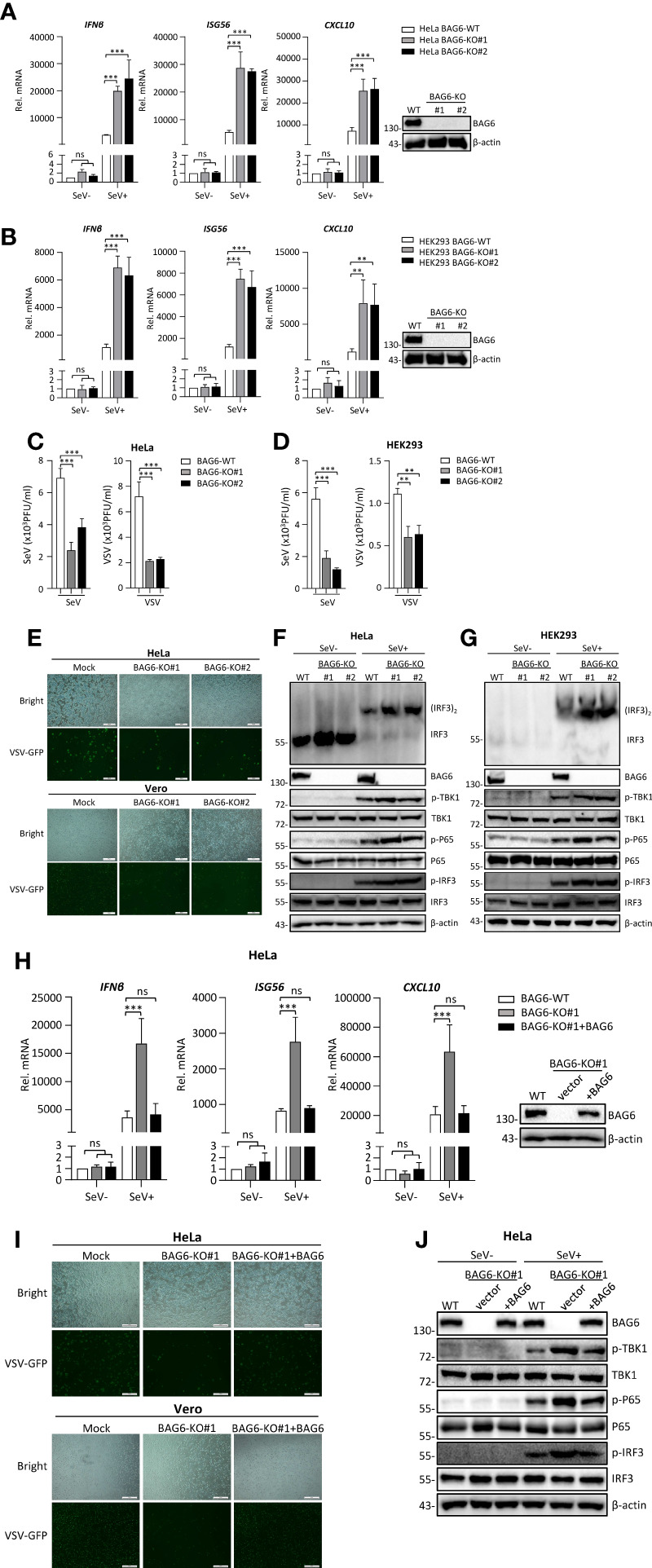
BAG6-deficiency significantly increases the transcription and activation of antiviral genes in HeLa and HEK 293 cells. **(A)** BAG6-deficiency enhances the transcription of downstream antiviral genes in HeLa cells. BAG6-deficient HeLa cells were left uninfected or infected with SeV (MOI = 1) for 8 h before qPCR analysis. **(B)** BAG6-deficiency enhances the transcription of downstream antiviral genes in HEK 293 cells. BAG6-deficient HEK 293 cells were left uninfected or infected with SeV (MOI = 1) for 8 h before qPCR analysis. **(C)** BAG6-deficiency inhibits the replication of SeV and VSV in HeLa cells. BAG6-deficiency HeLa cells were infected with SeV or VSV (MOI = 0.1) for twenty hours. The supernatants were collected for plaque assays to determine the viral titer. **(D)** BAG6-deficiency inhibits the replication of SeV and VSV in HEK 293 cells. BAG6-deficiency HEK 293 cells were infected with SeV or VSV (MOI = 0.1) for twenty hours. The supernatants were collected for plaque assays to determine the viral titer. **(E)** Effects of BAG6 deficiency on VSV-GFP replication. BAG6-deficient HeLa and control cells were infected with VSV-GFP (MOI = 0.1). The cells were viewed with Fluorescence Microscope after 16 h, and the supernatants were collected to infect Vero cells to determine the VSV-GFP replication. **(F, G)** BAG6 deficiency enhances RNA virus-induced dimerization of IRF3 and phosphorylation of TBK1, P65, and IRF3. BAG6-deficient HeLa and HEK 293 cells were uninfected or infected with SeV (MOI = 1) for 8 h before immunoblotting. **(H)** Reconstitution of BAG6 in BAG6-deficient HeLa cells suppresses virus-mediated innate immune responses. HeLa-BAG6-KO cells were transfected with control or BAG6 plasmids for 36 h. Cells then were left uninfected or infected with SeV (MOI = 1) for 8 h before qPCR analysis. **(I)** Reconstitution of BAG6 in BAG6-deficient HeLa cells enhances the replication of the virus. Similar to **(E)**, HeLa-BAG6-KO cells were transfected with control or BAG6 plasmids for 36 h, and cells then were infected with VSV-GFP (MOI = 0.1). **(J)** The experimental treatment was the same as **(H)** before immunoblotting. (**p < 0.01; ***p < 0.001; ns, no significant difference). The size bar represents 500 μm.

To investigate the effect of endogenous BAG6 on antiviral innate immune signaling, we generated BAG6-deficient monoclonal HeLa and HEK 293 cell lines by using the CRISPR Cas9-mediated gene knockout method. As shown in **Figures 3A, B**, we successfully knocked out the expression of BAG6 in HeLa and HEK 293 cells, and the qPCR analysis indicated the transcription level of *IFNβ, ISG56*, and *CXCL10* induced by SeV infection were significantly increased in BAG6-deficient HeLa and HEK 293 cells. Plaque assay showed that BAG6-deficiency in HeLa and HEK 293 cells inhibited the replication of SeV and VSV (**Figures 3C, D**). Furthermore, we found that the replication of GFP-tagged VSV was inhibited in BAG6-deficient HeLa cells compared with its wild-type cells (**Figure 3E**). The knockout of BAG6 in HeLa and HEK 293 cells dramatically promoted the IRF3 dimerization and the phosphorylation of TBK1, P65, and IRF3 after SeV infection (**Figures 3F, G**). However, the reconstitution of BAG6 in BAG6-deficient HeLa cells could turn its transcription of *IFNβ*, *ISG56*, and *CXCL10* genes induced by SeV back to a level similar to those in wild-type cells (**Figure 3H**). The reconstitution of BAG6 in BAG6-deficient HeLa cells could also turn the replication of GFP-tagged VSV back to a level similar to those in wild-type cells (**Figure 3I**). Further experiments found that the reconstitution of BAG6 in BAG6-deficient HeLa turned its potentiated phosphorylation of TBK1, P65, and IRF3 induced by SeV infection back to a level similar to those in wild-type cells (**Figure 3J**). These data suggest that BAG6 is a negative regulator of RNA virus-triggered signaling.

### The deficiency of BAG6 enhances SeV-mediated antiviral immune responses in primary mouse cells

**Figure 4:**
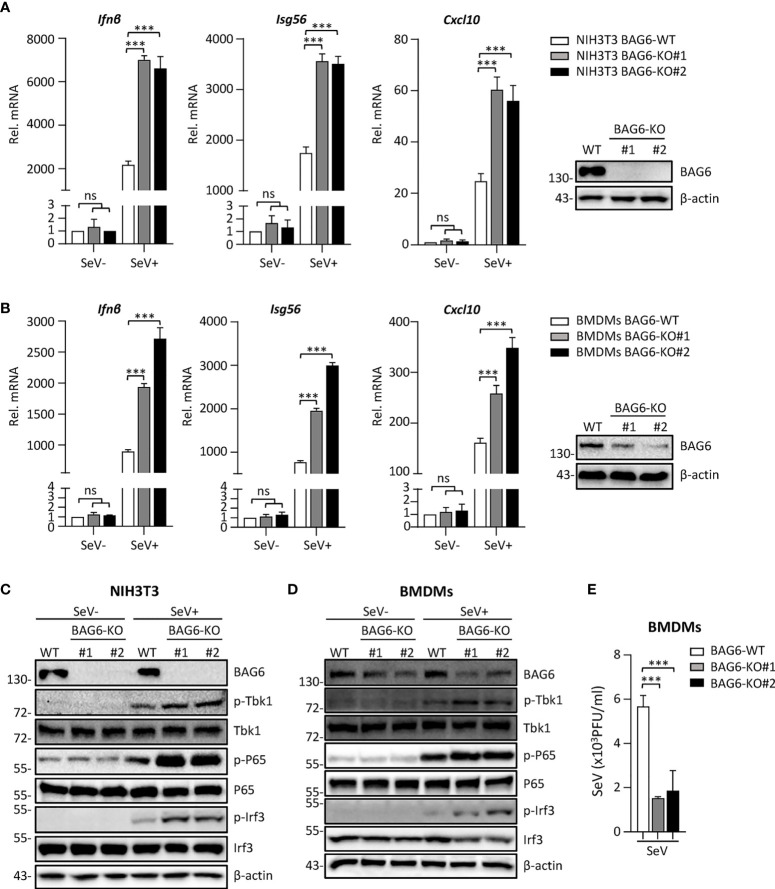
BAG6-deficiency significantly increases the RLR signaling in primary immune cells. **(A)** BAG6-deficiency enhances the transcription of downstream antiviral genes in NIH3T3 cells. BAG6-deficient NIH3T3 cells were left uninfected or infected with SeV (MOI = 1) for 8 h before qPCR analysis. **(B)** BAG6-deficiency enhances the transcription of downstream antiviral genes in mouse BMDMs. Mouse wild-type BMDMs (4×10^6^) were infected with lentivirus containing BAG6-CRISPR CAS9 sgRNA, the supernatants were replaced with fresh medium after 48 h, and the cells were infected with SeV (MOI = 1) for 8 h before qPCR analysis. **(C, D)** BAG6-deficiency enhances RNA virus-induced phosphorylation of TBK1, P65, and IRF3. BAG6-deficient NIH3T3 and BMDMs were untreated or treated with SeV for 8 h before immunoblotting. **(E)** Effects of BAG6-deficiency on virus replication. BAG6-deficient and wild-type BMDMs cells were infected with SeV (MOI = 0.1) for twenty hours. The supernatants were collected for plaque assays. (***p < 0.001; ns, no significant difference).

To further confirm the function of BAG6 in RLR-mediated signaling, we generated BAG6-deficient mixed primary mouse BMDMs cells by CRISPR Cas9-mediated gene knockout technology to assess the role of BAG6 in SeV-triggered signaling in primary mouse cells. We first verified that BAG6 was successfully knocked out in mouse NIH3T3 using the CRISPR-Cas9 method. Then, we performed a transient transfection using the CRISPR-Cas9 system to produce the BAG6-deficient primary mouse BMDMs cells. As shown in **Figures 4A, B**, the qPCR analysis showed that the SeV-induced transcription of *Ifnβ*, *Isg56*, and *Cxcl10* was significantly increased in BAG6-deficient NIH3T3 cells and primary mouse BMDMs. The knockout of BAG6 in NIH3T3 cells and mouse primary BMDMs cells dramatically promoted the phosphorylation of TBK1, P65, and IRF3 after SeV infection (**Figures 4C, D**). Furthermore, plaque assay showed that BAG6-deficient inhibited the replication of SeV in mouse primary BMDMs cells (**Figure 4E**). These experimental data suggest that BAG6 negatively regulates the RLR-mediated signaling in primary immune cells.

### BAG6 regulates the aggregation and recruitment of downstream signaling molecules of VISA

**Figure 5:**
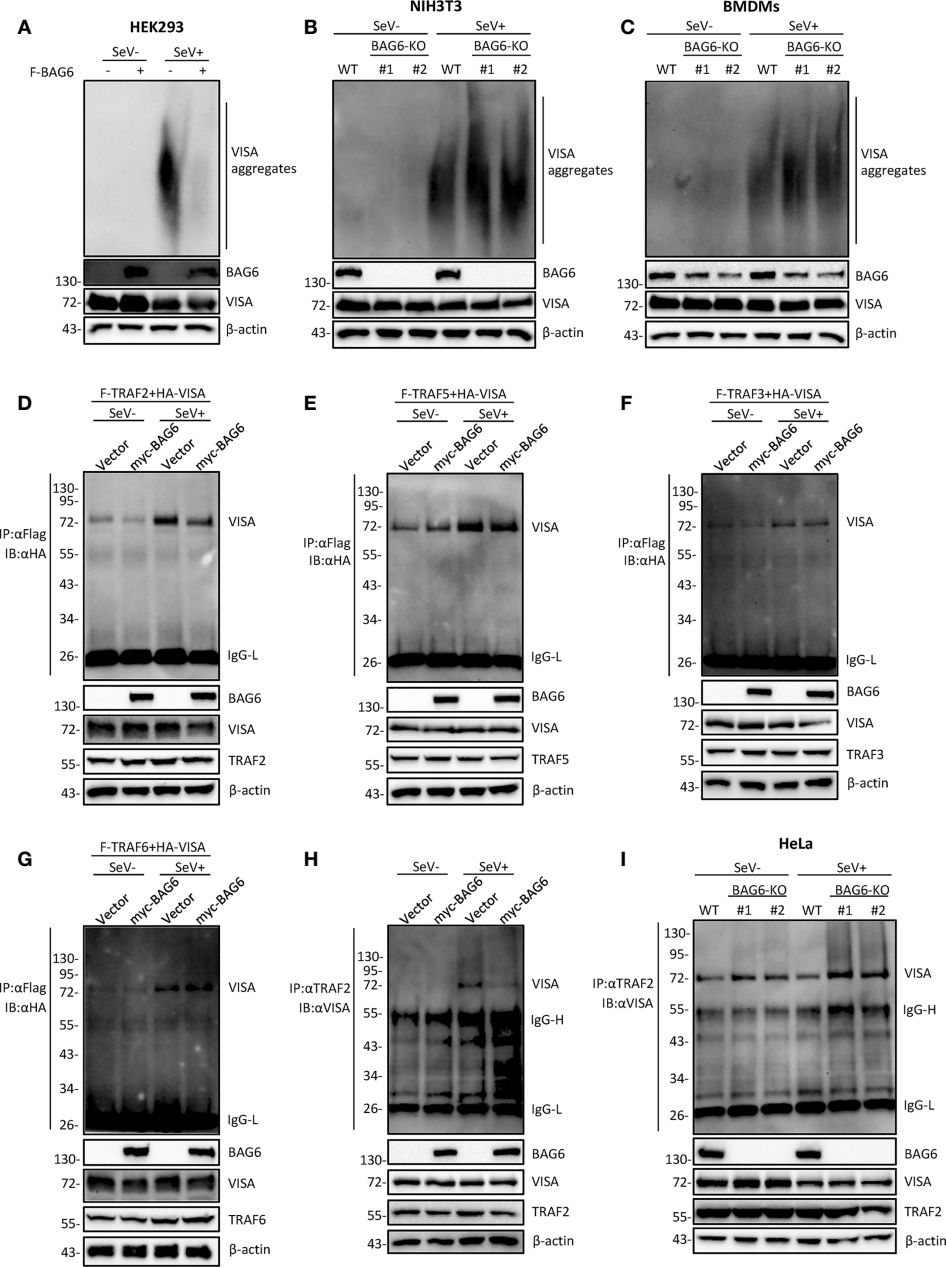
BAG6 regulates the aggregation and recruitment of downstream signaling molecules of VISA. **(A)** Overexpression of BAG6 inhibits SeV-induced aggregation of VISA. HEK 293 cells (2×10^6^) were transfected with vector or Flag-BAG6 for 20 h, and cells were infected with SeV (MOI = 1) for 8 h before harvest. Then, the lysates were fractionated by SDD-AGE and SDS-PAGE before immunoblotting analysis. **(B)** BAG6-deficiency increases SeV-induced aggregation of VISA in NIH3T3 cells. Wild type and BAG6-deficient NIH3T3 cells were untreated or treated with SeV (MOI = 1) for 8 h before harvest. Then, the lysates were fractionated by SDD-AGE and SDS-PAGE before immunoblotting analysis. **(C)** BAG6-deficiency increases SeV-induced aggregation of VISA in BMDMs. Wild-type BMDMs were infected with lentivirus containing BAG6-CRISPR Cas9 sgRNA for 48 h before viral infection. Wild type and BAG6-deficient BMDMs cells were untreated or treated with SeV (MOI = 1) for 8 h before harvest. Then, the lysates were fractionated by SDD-AGE and SDS-PAGE before immunoblotting analysis. **(D–G)** Overexpression of BAG6 inhibits the interaction of VISA and TRAF2 specifically. HEK 293 cells (2×10^5^) were transfected with the indicated plasmids for 22 h, and cells were untreated or treated with SeV (MOI = 1) for 10 h following co-immunoprecipitation and Western bolting analysis. **(H)** Overexpression of BAG6 impairs the endogenous interaction of VISA and TRAF2. HEK 293 cells (4×10^6^) were transfected with the indicated plasmids for 22 h, and cells were untreated or treated with SeV (MOI = 1) for 10 h following co-immunoprecipitation and Western bolting analysis. **(I)** BAG6-deficiency increases the endogenous interaction of VISA and TRAF2. Wild type and BAG6-deficient HeLa cells (1×10^7^) were untreated or treated with SeV (MOI = 1) for 10 h before co-immunoprecipitation and Western bolting analysis.

The aggregation of VISA is essential for its activation. Upon virus infection, VISA translocates to the outer membrane of mitochondria from cytoplasm and aggregates to form a giant prion-like aggregation complex which provides a platform for recruitment of downstream signaling molecules (11). We speculate whether BAG6 has an impact on the aggregation of VISA. The semi-denaturing detergent agarose gel electrophoresis (SDD-AGE) analysis indicated that overexpression of BAG6 extremely inhibited the aggregation of VISA in HEK 293 cells (**Figure 5A**), whereas BAG6-deficiency increased the SeV-triggered aggregation of VISA in NIH3T3 and primary mouse BMDMs cells (**Figures 5B, C**). These results indicate that BAG6 reduces antiviral response by inhibiting the aggregation of VISA. The activated VISA recruits downstream signaling molecules TRAFs, TBK1 and IKK complex. The results of the overexpression experiment in HEK 293 cells indicated that BAG6 inhibited the association of VISA with TRAF2 but not other TRAFs (**Figures 5D–G**). Endogenous co-immunoprecipitation results confirmed that the association between VISA and TRAF2 was hindered upon SeV infection (**Figure 5H**). Furthermore, BAG6-deficient significantly strengthened the association of VISA with TRAF2 in HeLa cells (**Figure 5I**). These results suggest that BAG6 inhibits innate immune signaling by attenuating the recruitment of TRAF2 to VISA.

### BAG6 potentiates the K48-linked ubiquitination of VISA/TRAF2

**Figure 6:**
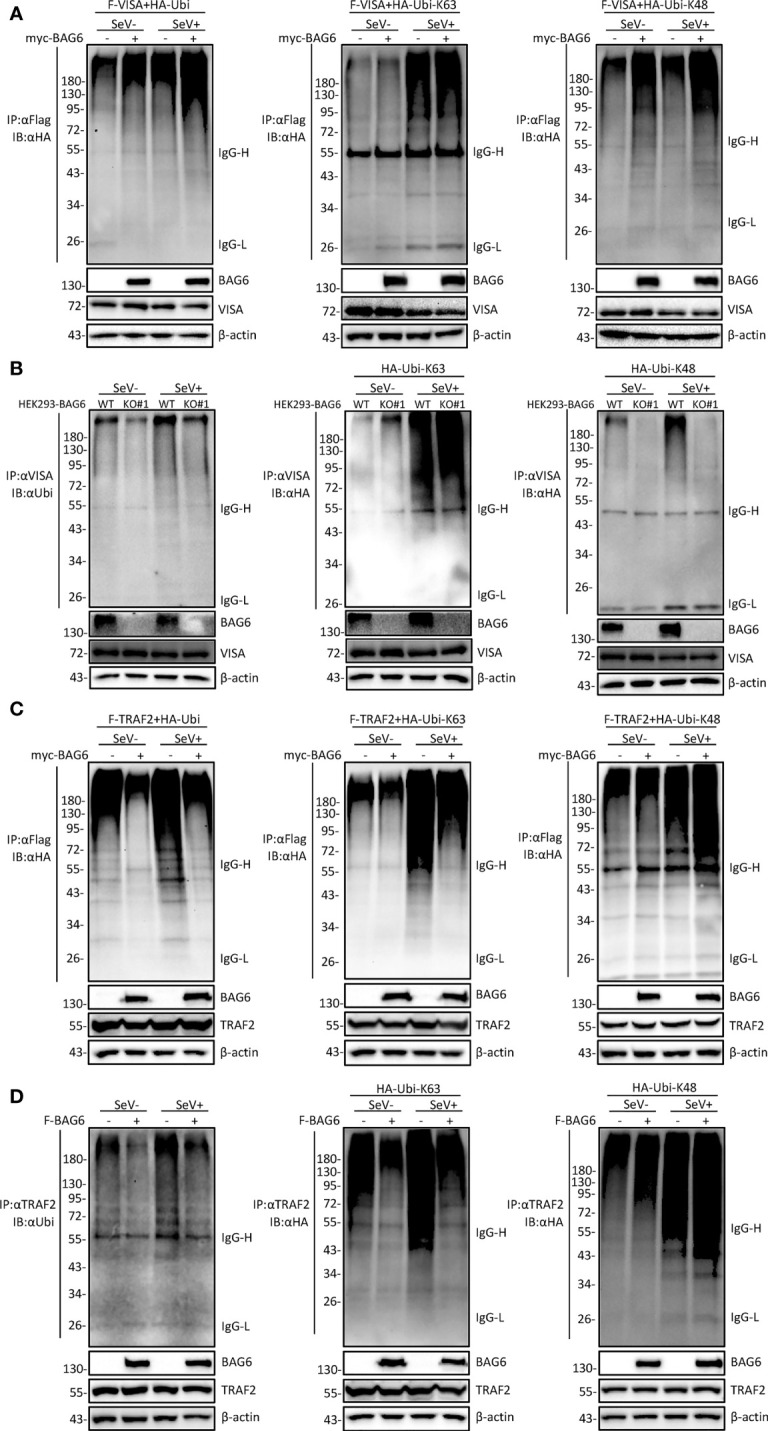
BAG6 promotes the K48-linked ubiquitination of VISA/TRAF2. **(A)** Overexpression of BAG6 increases the K48-linked polyubiquitin of VISA. HEK 293 cells (4×10^6^) were transfected with Flag-VISA, HA-ubiquitin (WT, K63R, or K48R), and Myc-BAG6 plasmids for 22 h, and cells were untreated or treated with SeV (MOI = 1) for 10 h before co-immunoprecipitation and Western bolting analysis. **(B)** BAG6-deficiency inhibits the K48-linked polyubiquitin of VISA. HEK 293 wild type and BAG6-deficient cells (4×10^6^) were transfected with HA-ubiquitin (K63R or K48R) for 22 h, and cells were untreated or treated with SeV (MOI = 1) for 10 h before co-immunoprecipitation and Western bolting analysis. **(C)** Overexpression of BAG6 increases the K48-linked polyubiquitin and impairs the K63-linked polyubiquitin of TRAF2. HEK 293 cells (4×10^6^) were transfected with Flag-TRAF2, HA-ubiquitin (WT, K63R, or K48R), and Myc-BAG6 plasmids for 22 h. Cells were untreated or treated with SeV (MOI = 1) for 10 h before co-immunoprecipitation and Western bolting analysis. **(D)** Overexpression of BAG6 increases the K48-linked polyubiquitin and impairs the K63-linked polyubiquitin of endogenous TRAF2. HEK 293 cells (4×10^6^) were transfected with HA-ubiquitin (WT, K63R, or K48R) and Flag-BAG6 plasmids for 22 h, and cells were untreated or treated with SeV (MOI = 1) for 10 h before co-immunoprecipitation and Western bolting analysis.

It has been reported that the polyubiquitination of VISA is essential for its activation (33, 34). To further investigate whether BAG6 affects the ubiquitination of VISA and TRAF2, co-immunoprecipitation experiments were performed, and the results showed that the overexpression of BAG6 in HEK 293 cells enhanced K48 but not K63-linked ubiquitination of VISA (**Figure 6A**), and the opposite results were obtained using the endogenous co-immunoprecipitation experiments in HEK 293-BAG6-deficient cells (**Figure 6B**). Further experiments showed that overexpression of BAG6 in HEK 293 cells enhanced K48-linked ubiquitination and inhibited K63-linked ubiquitination of TRAF2, ultimately leading to an increase in total ubiquitination (**Figure 6C**), and the consistent results were obtained using the endogenous co-immunoprecipitation experiments (**Figure 6D**). These data indicate that BAG6 may promote the K48-linked ubiquitination of VISA and TRAF2, reducing VISA aggregation and further reducing the recruitment of TRAF2 to regulate RLR-mediated antiviral signal transduction negatively.

## Discussion

The RLRs (RIG-I-like receptors) in the cytoplasm, including RIG-I and MDA5, recognize viral RNA. This viral RNA binding leads to a conformational change of the RLRs, triggering their oligomerization and activation. The activated oligomerization of RLRs provides a template of the oligomer to initiate the assembly of adaptor protein VISA on the outer membrane of mitochondria, leading to VISA’s activation. Once VISA is aggregated and activated, the downstream signaling molecules, including the kinase IKKs, and TBK1, are recruited to the aggregation platform, leading to the activation of IRF3/IRF7 and NF-κB to turn on the production of interferons and proinflammatory cytokines. A few E3 ubiquitin ligases, including TRAF2, TRAF3, TRAF5, and TRAF6, are recruited by VISA through their specific binding motif. The K63 and K48-linked polyubiquitination modification in these signaling cascades are essential for VISA downstream signaling (35). Our study demonstrates the critical role of BAG6 in the innate immune response by promoting the K48-linked ubiquitination of VISA, resulting in a weakened aggregation of VISA.

BAG6 is a ubiquitin-like protein shuttling between nuclear and cytoplasm involved in apoptosis, T-cell response, and antigen presentation (20). It interacts with HSC70 protein and recognizes the misfolded protein, allowing their proteasomal degradation (20). BAG6 is critical for regulating autophagy by modulating EP300/p300 intracellular location (36). It was reported that BAG6 associates with mitofusin1 and 2, localizing on mitochondria, and has a physiological function in mitochondrial fission (37).

Previously, we identified several regulatory proteins (including TARBP2 (31), RACK1 (38), HSPBP1 (39), SNX5 (40), DUT (41) and N4BP3 (42)) involved in the RIG-I/VISA signaling pathway. Recently, our experiments suggest that BAG6 is crucial in negatively regulating RLRs-VISA-mediated antiviral signaling. Overexpression of BAG6 hampered SeV-triggered activation of IFN-β promoter, ISRE, and NF-κB. BAG6-deficiency greatly enhanced the transcription of *IFNβ*, *ISG56*, and *CXCL10* induced by SeV infection. Further experimental results showed that overexpression of BAG6 inhibited SeV-induced phosphorylation of TBK1, P65, and IRF3. In contrast, the opposite results were obtained in BAG6-deficiency cells (HEK 293, HeLa, NIH3T3, and primary mouse BMDMs).

Our experimental results also showed that overexpression of BAG6 inhibited RIG-I-N and VISA-mediated, but not TBK1 or IRF3-5D-mediated activation of IFN-β promoter and ISRE, indicating that BAG6 inhibits RLRs-VISA-mediated signaling by targeting VISA. Consistently, BAG6 interacted with VISA specifically, and the interaction between BAG6 and VISA was enhanced upon SeV infection.

Virus infection-induced VISA aggregation is critical for downstream antiviral signaling activation (11, 13). Our experimental results demonstrated that BAG6 inhibited the aggregation of VISA induced by SeV infection. The further co-immunoprecipitation experiment showed that recruitment of TRAF2 to VISA is hindered. Previous studies have shown that the aggregation of VISA could be robustly induced *in vitro* by incubating mitochondria with RIG-I and K63 ubiquitin chains (11, 13), which suggests that K63 ubiquitination of VISA is important for its activation. The analysis of the results of ubiquitination experiments indicated that BAG6 enhanced K48-linked ubiquitination of VISA but had no effect on K63-linked ubiquitination. It has been confirmed that PINK1 inhibits aggregation and signaling of VISA (19), and BAG6 promotes the PINK1 signaling pathway (26). Perhaps BAG6 and PINK1 have a synergistic effect on inhibiting the aggregation of VISA. BAG6 facilitated the K48-linked ubiquitination of VISA may lead to inhibition of signal transduction by a failure of VISA to aggregate normally after virus Infection. However, BAG6 is not an E3 ubiquitin ligase; according to current reports, E3 ubiquitin ligases-mediated (RNF5, RNF125, MARCH5, TRIM25, AIP4) K48-linked ubiquitination initiates proteasomal degradation of VISA (43, 44). Moreover, it’s reported that TRIM29 induced ubiquitination of Lys371, Lys420 and Lys500 sites on VISA and degradation of VISA through K11-linked polyubiquitination (45). TRIM29 was also found to promote K48 ubiquitination and degradation of NEMO to suppress the innate immune response (46). These evidences suggest that TRIM29 is an important E3 ligase in the negative regulation of VISA-mediated signaling pathways. BAG6 correlates well with E3 ubiquitin ligases RNF126 and FBXO7-SCF. Whether BAG6 is involved in regulating the ubiquitination of VISA through them or the reported E3 ubiquitin ligases remains to be investigated. Besides, BAG6 inhibited K63-linked and promoted K48-linked ubiquitination of TRAF2. Previous studies have shown that phosphorylation and K63-linked ubiquitination of TRAF2 is important for signaling (47–50).

TRAFs, critical proteins involved in VISA-mediated signaling, are essential for RNA virus-induced IRF3 activation and IFNβ production. The current studies have shown that VISA-mediated innate immune activation is dependent on TRAFs and partially on NEMO, but not on TBK1 binding proteins (51). When TRAF2/3/5/6 were compounded *via* CRISPR Cas9 knockout, cells absolutely lost the RNA virus response, suggesting that TRAFs are essential in VISA-mediated signal transduction (51, 52). When a single TRAF was knockout, type I interferon levels in TRAF2^-/-^ and TRAF6^-/-^ cells were decreased substantially, while type I interferon levels in TRAF3^-/-^ and TRAF5^-/-^ cells remained normally induced (51). TRAF2, TRAF3, TRAF5, and TRAF6 were reconstituted in cells with TRAF2/3/5/6 compound knockout, respectively, and cells with reintroduced TRAF6 had the strongest type I interferon production, while TRAF5 had the lowest activity, these results suggest that TRAF6 is essential, while TRAF5 is dispensable (51). Another study showed that IRF3 phosphorylation was extensively lost when TRAF2 and TRAF5 were double knockout, and IRF3 phosphorylation could be induced normally when TRAF2 or TRAF5 were knockout alone (52). Furthermore, after reconstitution of TRAF2 or TRAF5 in TRAF2 and TRAF5 double knockout cells, TRAF2 reintroduction led to a basic restoration of IRF3 phosphorylation levels. In contrast, TRAF5-induced IRF3 phosphorylation level was low, suggesting that TRAF2 is a lot greater essential than TRAF5 in the signaling process and that TRAF5 may play a partly regulatory role in VISA-mediated signaling but is not required (52). Our experimental results show that BAG6 hinders the signal transduction of VISA and downstream TRAF2 but not TRAF5 by interacting with VISA, which is consistent with the above findings.

In conclusion, our results indicate that BAG6 suppresses RNA virus-mediated innate immunity by promoting K48-linked ubiquitination of VISA/TRAF2 and inhibiting VISA aggregation, further inhibiting the recruitment of downstream components reduced the transcript levels of downstream antiviral genes.

## Data availability statement

The original contributions presented in the study are included in the article/supplementary material. Further inquiries can be directed to the corresponding author.

## Ethics statement

The animal study was reviewed and approved by the Animal Care Committee of Jiangxi Normal University College of Life Sciences.

## Author contributions

J-PH: Investigation, formal analysis, writing-original draft. JL: Validation, formal analysis. Y-PX: Validation. L-GX: Supervision, writing-original draft, writing-review and editing, funding acquisition, conceptualization, methodology. All authors read, and approved the submitted version. All authors contributed to the article and approved the submitted version.

## Funding

This work was supported by the National Natural Science Foundation of China (Grant Nos. 81971502, 82060298).

## Acknowledgments

We sincerely thank Dr. Hong-Bing Shu (Medical Research Institute, Wuhan University) for providing plasmids and other reagents. We thank Jiangxi Normal University, College of Chemistry and Chemical Engineering for help with the Laser Confocal Microscope analysis.

## Conflict of interest

The authors declare that the research was conducted in the absence of any commercial or financial relationships that could be construed as a potential conflict of interest.

## Publisher’s note

All claims expressed in this article are solely those of the authors and do not necessarily represent those of their affiliated organizations, or those of the publisher, the editors and the reviewers. Any product that may be evaluated in this article, or claim that may be made by its manufacturer, is not guaranteed or endorsed by the publisher.
